# Assessment of the core and support functions of the Integrated Disease Surveillance system in Maharashtra, India

**DOI:** 10.1186/1471-2458-13-575

**Published:** 2013-06-13

**Authors:** Revati K Phalkey, Sharvari Shukla, Savita Shardul, Nutan Ashtekar, Sapna Valsa, Pradip Awate, Michael Marx

**Affiliations:** 1 Institute of Public Health (Former Department of Tropical Hygiene and Public Health) Im Neuenheimer Feld 324, University of Heidelberg, Heidelberg, Germany D-69120; 2 Centre for Modelling and Simulation, University of Pune, Pune, Maharashtra, India; 3 State Surveillance Office, Integrated Disease Surveillance Project, Ministry of Health and Family Welfare, Pune, Maharashtra, India; 4 Chest Research Foundation, Pune, Maharashtra, India

**Keywords:** Integrated Disease Surveillance and Response (IDSR), Assessment, Core and support surveillance functions, Maharashtra, India

## Abstract

**Background:**

Monitoring the progress of the Integrated Disease Surveillance (IDS) strategy is an important component to ensure its sustainability in the state of Maharashtra in India. The purpose of the study was to document the baseline performance of the system on its core and support functions and to understand the challenges for its transition from an externally funded “project” to a state owned surveillance “program”.

**Methods:**

Multi-centre, retrospective cross-sectional evaluation study to assess the structure, core and support surveillance functions using modified WHO generic questionnaires. All 34 districts in the state and randomly identified 46 facilities and 25 labs were included in the study.

**Results:**

Case definitions were rarely used at the periphery. Limited laboratory capacity at all levels compromised case and outbreak confirmation. Only 53% districts could confirm all priority diseases. Stool sample processing was the weakest at the periphery. Availability of transport media, trained staff, and rapid diagnostic tests were main challenges at the periphery. Data analysis was weak at both district and facility levels. Outbreak thresholds were better understood at facility level (59%) than at the district (18%). None of the outbreak indicator targets were met and submission of final outbreak report was the weakest. Feedback and training was significantly better (p < 0.0001) at district level (65%; 76%) than at facility level (15%; 37%). Supervision was better at the facility level (37%) than at district (18%) and so were coordination, communication and logistic resources. Contractual part time positions, administrative delays in recruitment, and vacancies (30%) were main human resource issues that hampered system performance.

**Conclusions:**

Significant progress has been made in the core and support surveillance functions in Maharashtra, however some challenges exist. Support functions (laboratory, transport and communication equipment, training, supervision, human and other resources) are particularly weak at the district level. Structural integration and establishing permanent state and district surveillance officer positions will ensure leadership; improve performance; support continuity; and offer sustainability to the program. Institutionalizing the integrated disease surveillance strategy through skills based personnel development and infrastructure strengthening at district levels is the only way to avoid it from ending up isolated! Improving surveillance quality should be the next on agenda for the state.

## Background

Epidemiologic surveillance is the “on going systemic collection, analysis, and interpretation of health data essential to the planning, implementation, and evaluation of public health practice, closely integrated with the timely dissemination of these data to those who need to know. The final link in the surveillance chain is the application of these data to prevention and control. A surveillance system includes a functional capacity for data collection, analysis, and dissemination linked to public health programs” [1]. Today, surveillance attains importance more than ever given the stark reductions in travel times that essentially catalyze the spread of emerging pathogens and the introduction of existing ones to new areas. Present day surveillance systems chase moving targets in terms of both evolving pathogens and adapting hosts, in the backdrop of rapidly changing social and economic human environments [2].

### Strategies in communicable disease surveillance: single - vertical vs. multiple integrated

Surveillance is largely interpreted and implemented as “a component” within vertical single disease control programs in most low and middle income countries including India [3]. Especially because not all countries are able to allocate resources for surveillance and decision makers often hesitate to prioritize outbreak detection when curative needs are barely met [4]. Evidence suggests that heavily centralized, vertical single disease control programs although resource (personnel, equipment, and financial) intensive tend to be effective and are well implemented [5]. Nonetheless, several drawbacks have been identified with these programs and chief amongst them being their inability to fulfill surveillance functions adequately. Majority of them are heavily centralized and offer sub-optimal speed of surveillance, largely inadequate to detect outbreaks in a timely manner [6,7]. Duplication of efforts across multiple un-coordinated single disease control programs overburdens sub-national staff and are a waste of valuable resources [8,9]. Last but not the least, vertical programs are heavily autonomous in their functioning and often non-flexible, preventing their integration at any level and making them unsustainable and unaffordable in the long run [10].

Failure of vertical single disease control strategies to overcome the global burden of communicable diseases despite the monetary investments and the emergence of newer challenges to disease control, prompted the WHO to develop and advocate an Integrated approach to Disease Surveillance and Response (IDSR) in 1998 [11]. Integrated disease surveillance is “a combination of active and passive systems that use a single infrastructure to gather information about multiple diseases or behaviours of interest using similar structures, personnel and processes” [12]. The strategy aims to “strengthen surveillance and response at each level of the health system by building local capacities; leveraging strengths and expertise through partnerships and coordination; training personnel at all levels; mobilizing resources; integrating existing multiple disease-specific surveillance systems to ensure efficient use of resources; improve the use and flow of surveillance information; strengthen laboratory capacity; emphasize community and clinician participation; use data thresholds to trigger alerts and above all link surveillance; laboratory confirmation; and other data to public health actions” [13-15]. IDSR “recognizes that different diseases have specialized surveillance needs, and exploits opportunities for synergies in carrying out core and support functions” [16]. However, the capability of existing disease control programs to complement each other eventually determines the success of this approach.

Forty six member states of the WHO-AFRO have adopted the integrated disease surveillance strategy since 1998. As of 2010, 86% of the 4386 districts in 45 countries in the region have implemented IDSR in one form or the other [17]. A systematic review of evidence from 18 countries indicates mixed experiences [18]. Limited information is available on the number and status of countries adopting it in the WHO SEARO region including in India, although a regional strategic plan (2002–2010) for integrated disease surveillance was developed and adopted in 2002 [19].

### Disease burden and the Integrated approach to communicable Disease Surveillance (IDS) in India

Like most other lower-middle income countries, India has to brave its path through the daunting triple burden of communicable diseases (the old elephant), chronic non-communicable diseases (the emerging dinosaur) and weak overburdened health care system largely incapable of combating the two simultaneously [20,21]. With a population of over 1.21 billion and a decadal growth rate of 15.9%, by sheer numbers India bears a large part of the global burden of communicable disease and potential hosts [10]. Infectious diseases contribute to about 26% of the disease burden in India [22]. Together with maternal, perinatal and nutritional disorders, infectious diseases constitute 38% of the total deaths (predominantly in rural areas) [23]. Additionally emerging infections like Gp-B rota virus, nipah virus, and chandipura virus pose separate challenges [24]. Although disease trends for poliomyelitis, tuberculosis, neonatal tetanus, measles and HIV and AIDS in the country are decreasing, those for dengue, chikungunya, HIV and TB co-infections, cholera 0139, japanese encephalitis, leptospirosis and novel H1N1 infections are on the rise [23].

India adopted the Integrated Disease Surveillance (IDS) strategy in 2004 following the limited success and eventual discontinuation of the National Surveillance Program for Communicable Diseases (NSPCD 1997–2002) [14]. In phase one, nine pilot states implemented the strategy through the World Bank funded Integrated Disease Surveillance Project (IDSP), 2005–2012. The strategy was expanded to all 29 states in a phased out manner under the National Rural Health Mission (NRHM), funded by core domestic state budgets [25].

The IDSP in India is a three-tier decentralized state based system, covering 13 mandatory diseases and syndromes with a focus on the districts (Additional file 1). Additionally states are allowed to add up to five state specific conditions for surveillance [26]. The National (NSU), State (SSU) and District Surveillance Units (DSU) are the organizational structures, each headed by a surveillance officer and supported by epidemiologists, microbiologists, data entry operators and data managers. There are over 604 DSUs in the country, 35 SSU- one each at the respective state headquarters which directly report to the NSU based at the National Centre for Disease Control (NCDC former National Institute for Communicable Diseases NICD) in Delhi [27].

**Figure 1:**
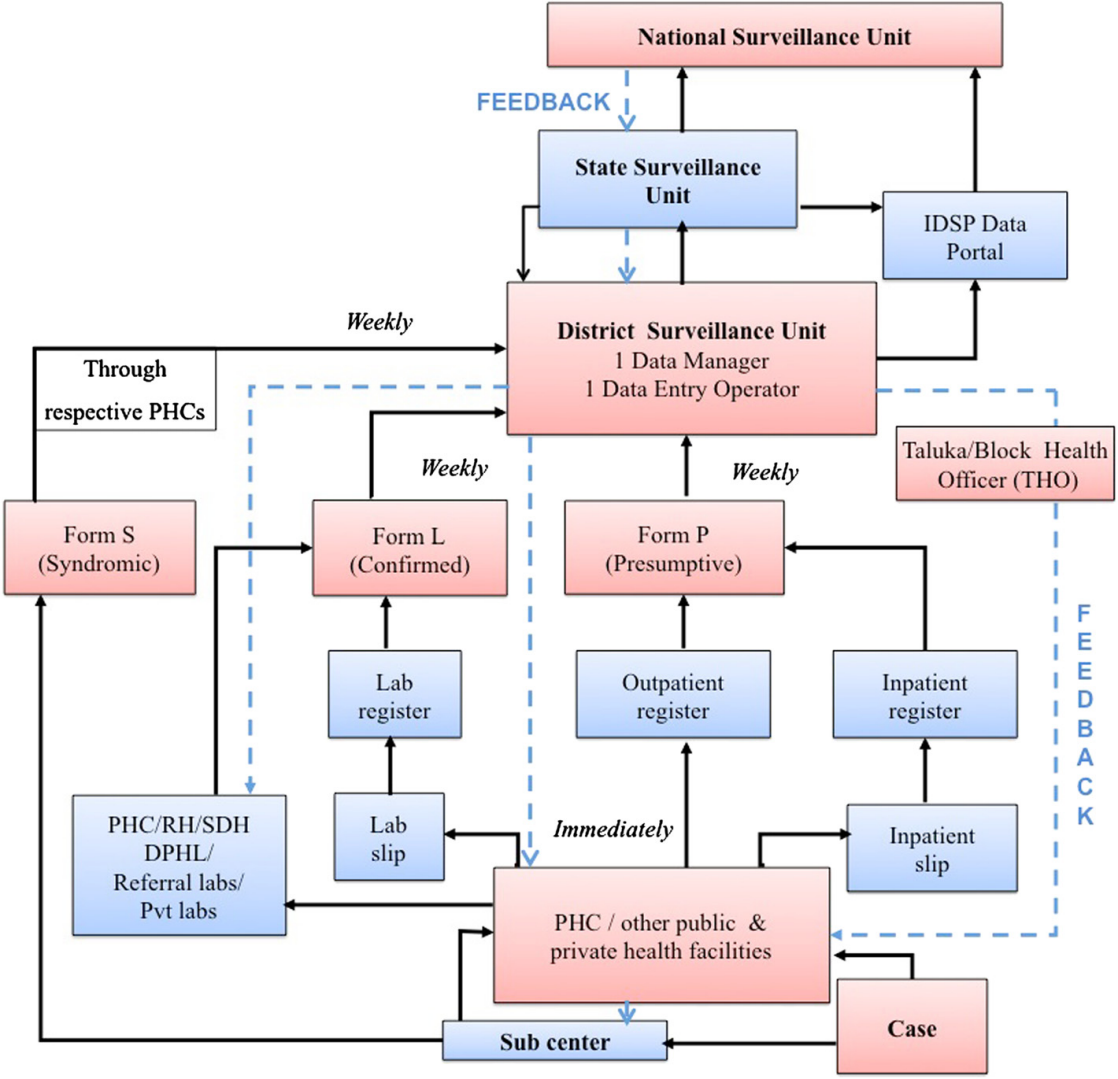
**Organizational structure of the IDSP system in Maharashtra**, **India.**

IDSP uses syndromic, presumptive and lab confirmation approaches to collect data on cases of priority diseases through S, P and L reporting formats respectively from identified public and private reporting units on a weekly basis in both urban and rural areas as shown in Figure 1. Data is collected on paper based forms up to the DSU level where it is electronically transferred to the state and central levels through the IDSP portal and via Email [28].

The study aimed to assess the structure and performance of the Integrated Disease Surveillance System in the Maharashtra state of India, which was a consistent pilot state for both of the only two national level surveillance efforts in the country - namely the NSPCD and the IDSP. Infectious diseases contribute to over 50% burden of disease in the state [29]. Since IDSP was implemented in Maharashtra in 2005 it was in its early stages of independent operation in 2011 and assessing its progress was important to ensure its sustainability. Documenting the baseline performance was necessary for future comparisons. The World Bank funding was to conclude in March 2012 in all nine pilot states including Maharashtra. Therefore, IDSP was in transition from an externally funded “project” to a state owned state run surveillance “program” for the first time- mandating a review of the lessons learned. The study also aimed to understand the challenges for successful integration of surveillance functions in the district health care machinery and to make recommendations for a smooth transition.

## Methods

### Study area

**Figure 2:**
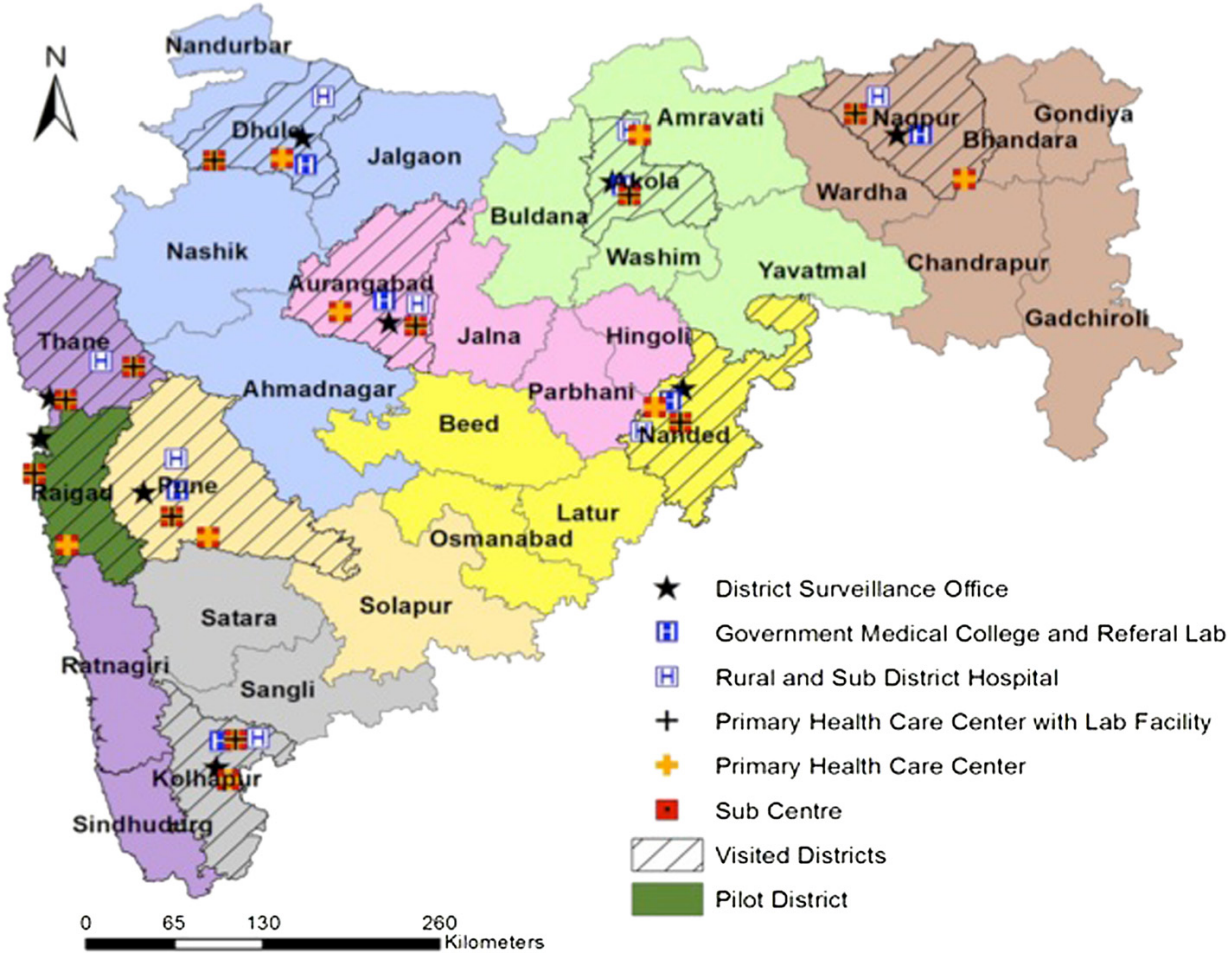
Study area.

Maharashtra, the second largest state in India covers a population of 112 million and is organized administratively into eight health circles covering 33 districts and a large Mumbai urban metropolitan area (Figure 2). All 34 (100%) DSUs were included in the survey at the state level. An onsite visit was conducted in ten (30%) randomly identified districts with care to include at least one district from each of the eight health circles. Mumbai being the only urban unit was purposively sampled.

### Study design and sampling

**Figure 3:**
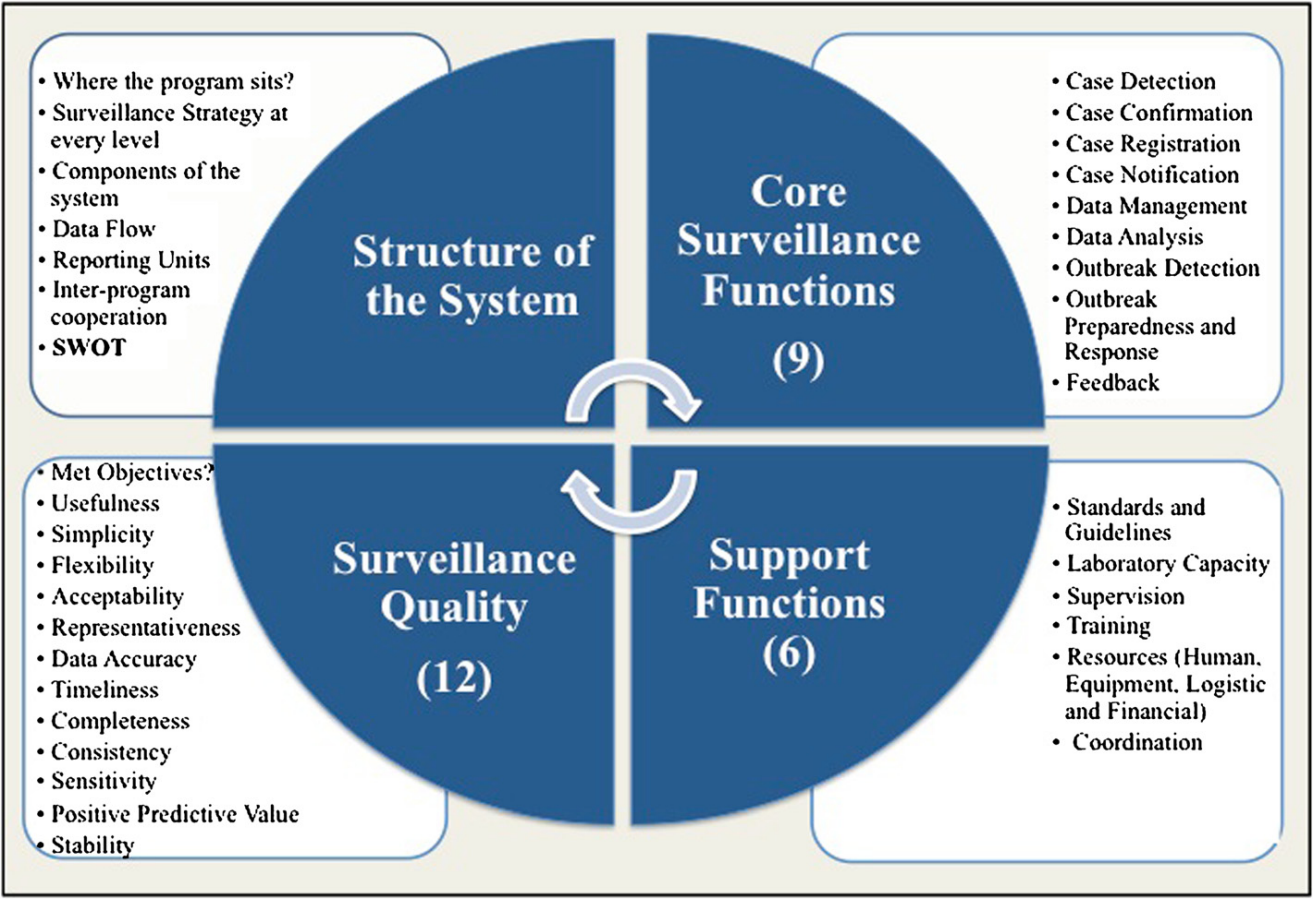
**Scope of the assessment study (Source modified from[**[34]**]).**

Multi-centre, retrospective cross-sectional evaluation study was conducted along four key areas namely- objectives, structure and components of the system; core surveillance functions; support surveillance functions and quality attributes. Modified versions of the WHO protocol for assessing national surveillance systems [30]; WHO guide to assessing disease surveillance and response systems [31]; CDC 2001 updated guidelines [32] and the 2004 framework [33] for evaluating public health surveillance systems; and the WHO framework for evaluating communicable disease surveillance systems [34] were used to design the study protocols (Figure 3). Core functions included nine aspects namely case detection; case registration; case confirmation; case notification; data management; data analysis; outbreak preparedness; outbreak response; and feedback. Support functions included six aspects namely manuals and guidelines, laboratory capacity; supervision; training; resources (financial, human, material and equipment) and coordination. Results of the system’s attributes are described elsewhere [18].

Multi-stage simple random sampling was done. At each of the ten districts a list of all S, P and L Reporting Units (RUs) was obtained and the facilities were ranked based on the reproductive and child health indicators. Of the top five ranking facilities, one Rural (RH) or Sub-District Hospital (SDH), two Primary Health care Centres (PHCs) were randomly identified. Once at the PHC, one subcenter was randomly identified for a visit. A Government Medical College (GMC) hospital was included in the study when available. A total of 46 facilities (6 GMCs, 8 RHs, 16 PHCs and 16 subcenters); and 25 laboratories (10 PHC; 8 RH and 7 referral laboratories (RL)) were visited.

### Data collection

Secondary data included record review at the state, district, facility and laboratory levels particularly on the IDSP data portal, print manuals, official correspondence and reports, weekly S, P and L forms, results of data analysis, meeting schedules, minutes, training schedules, feedback bulletins, outbreak investigations and response, annual audit reports, daily diaries at subcenters, outpatient, inpatient and laboratory registers at facilities. Primary data was collected through structured interviews administered in person in the ten districts and electronically via Email (followed up by a telephone call) in 24 districts after obtaining consent. Feedback was provided to each district via Email. Results of the primary data analyses were presented to the state and 34 district surveillance officers for validation.

### Data analysis

Primary and secondary data were entered, cleaned and analyzed in Microsoft™ Excel® 2010. Frequency distributions were calculated for all variables and identified indicators. Data from the structured questionnaires were then imported in SPSS® version 17 for univariate and bivariate analysis. Mann–Whitney test was performed on responses from the electronic and visited district questionnaires and no significant differences were observed.

### Study limitations

The study was conducted in collaboration with the SSU. In order to counter the fear of disciplinary action if deficiencies were identified, the respondents were assured of anonymity and confidentiality. The focus of the assessment was on lesson learning and constructive criticism which was highlighted before every interview with support from the SSU.

### Ethics approval

The study was a part of the doctoral dissertation of the lead author and was passed through the ethical committee of the Medical Faculty, University of Heidelberg, Germany (Approval Number: S381/2010) and by the University of Delhi, India (Approval Number: Anth/2009/585). In addition relevant clearances were also obtained from the Ministry of Health and Family Welfare and the National Institute of Communicable Diseases to conduct the assessment.

## Results

### Assessment of the structure of IDSP

A review of the current structure of IDSP against the original project implementation plans at the national and state levels revealed that several aspects have been modified over the last seven years. Chief amongst them were:

•Lab structure changed from L1 (primary), L2 (secondary) and L3 (tertiary) to L reporting units.

•Referral lab structure (2010) with ten identified government teaching hospital labs

•Strengthening of the District Public Health Laboratories (DPHL) for routine surveillance not done

•Modification of conditions under surveillance- TB and HIV, traffic accidents and air pollution were removed and H1N1 and H5N1 added.

•The reporting formats have been changed thrice in the life of the project

•Two additional office assistant positions were eliminated at state level

•One week grace period provided for portal data entry

•State surveillance unit was non-functional from June 2008 to June 2009

•No annual trainings conducted despite 8% of total state budget earmarked

•No mandatory supervisory strategy in spite of quarterly provision in original plans

•Trigger levels for outbreak response revised from 1–3 to 1–5

### Core functions

Tables 1 and 2 provide an overview of the inter-facility and facility- district differences in core and support function performance of the IDSP in the state.

**Table 1:** Assessment of the core surveillance functions [n (%)]

	**Subcenter (16)**	**PHC (16)**	**GMC (6)**	**RH (8)**	**Facility Total (46)**	**District Total (34)**
**Case Detection**						
Availability of Standard Case Definitions (SCDs)	10 (62)	10 (62)	1 (17)	3 (37)	**24** (**52**)	**29** (**85**)
SCDs stated correctly	16 (100)	13 (81)	2 (33)	7 (87)	**38** (**82**)	-
**Case Registration**						
Complete IPD registers	-	10 (62)	3 (50)	5 (62)	**18** (**39**)	-
Complete OPD registers	10 (62)	10 (62)	2 (33)	2 (25)	**14** (**30**)	-
**Case Confirmation**/ **Notification**						
Capacity to transport specimens to higher lab	12 (75)	14 (87)	5 (83)	8 (100)	**39** (**85**)	**29** (**85**)
Lack of one or more of six reporting forms	13 (81) *S forms only*	7 (44)	2 (33)	4 (50)	**13** (**28**)	**13** (**38**)
Correct definition of “reporting week”	2 (12)	3 (19)	2 (33)	0 (0)	**7** (**14**)	**15** (**44**)
**Data Analysis**						
Summarize and present data in a table	2 (12)	4 (25)	4 (67)	1 (12)	**11** (**24**)	**34** (**100**)
Perform trend analysis (regular data)	2 (12)	3 (19)	1 (17)	0 (0)	**6** (**13**)	**28** (**82**)
Calculate incidence and prevalence of diseases	0 (0)	3 (19)	0 (0)	0 (0)	**3** (**6**)	**17** (**50**)
Availability of accurate denominators	12 (75)	16 (100)	2 (33)	3 (37)	**33** (**72**)	**26** (**76**)
**Outbreak Detection**						
Compare present and previous data	14 (88)	10 (62)	1 (17)	3 (37)	**28** (**61**)	**27** (**80**)
Have an action threshold for the state’s priority diseases	9 (56)	15 (94)	4 (67)	6 (75)	**34** (**74**)	**14** (**41**)
Stated correct threshold (Measles, Cholera, Diphtheria)	8 (50)	10 (62)	3 (50)	2 (25)	**23** (**50**)	**6** (**18**)
**Outbreak Response**						
Manual for standard case management	12 (75)	14 (87)	4 (67)	7 (87)	**37** (**80**)	**21** (**62**)
Prevention and control measures based on local data	16 (100)	16 (100)	2 (33)	7 (87)	**41** (**89**)	**20** (**59**)
**Received Feedback**	2 (12)	2 (12)	1 (17)	2 (25)	**7** (**15**)	**22**(**65**)

**Table 2:** **Assessment of the support surveillance functions** [n (%)]

	**Subcenter (16)**	**PHC (16)**	**GMC (6)**	**RH (8)**	**Facility Total (46)**	**District Total (34)**
**Availability of technical guidelines and manuals**	3 (19)	3 (19)	0 (0)	1 (12)	**7** (**15**)	**23** (**67**)
**Supervision**	7(44)	7(44)	2 (33)	1 (12)	**17** (**37**)	**6** (**18**)
**Training**	6 (37)	6 (37)	2 (33)	3 (37)	**17** (**37**)	**26** (**76**)
**Coordination**						
Coordination mechanism/ body	10 (62)	13 (81)	3 (50)	5 (62)	**31** (**67**)	**22** (**65**)
IDSP focal person	13 (81)	14 (87)	6 (100)	8 (100)	**41** (**89**)	**30** (**88**)

#### Case detection and registration

Standard case definitions were available in English only and were regularly used by thirty one (67%) facilities. There were no IDSP registers at subcenters but records of patients attended were maintained in a daily diary. All other 30 facilities maintained outpatient (OPD) and inpatient (IPD) registers and 29 (97%) produced them for inspection. IPD registers were more complete than OPD registers. Registers were incomplete in larger facilities particularly with respect to the diagnosis and address of the patient reportedly due to the high volume of patients and unavailability of computer literate staff.

#### Case confirmation

At the district level, thirty (88%) districts maintained a documented list of referral laboratories and twenty nine (85%) acknowledged a mechanism for timely referral of samples. Eighteen (53%) districts had laboratory capacity to confirm all identified diseases. Others referred samples to higher public laboratories, or in cases of emergencies, used private labs. Six of the 16 PHCs visited did not have a lab. All 46 facilities (100%) performed blood smear examination for malaria, 36 (78%) performed Ziehl Neelson stain for sputum, and 27 (59%) stool for microscopy. Cerebro Spinal Fluid (CSF) examination for meningitis was performed only at the referral labs due to lack of qualified staff and availability of appropriate equipment at periphery. Capacity of the facilities to handle samples until shipment varied and was better for blood (93%), than for sputum (74%), and stool (41%) samples. Majority of the facilities referred the patient rather than sending samples due to unavailability of functional cold chain (71%) and appropriate transport media (50%). Thirteen (38%) districts and seven (15%) facilities reported delays in case and outbreak detections either due to lack of trained laboratory staff or lack of equipment and reagents.

#### Case notification

Majority of the districts (33, 97%) received data hand delivered in paper based formats. Twenty-three (68%) districts additionally accepted verbal reporting via mobile/cell phones without a formal mechanism to document it. All DSUs transmitted data to the state via Email or the IDSP portal. Ten (29%) districts experienced regular problems with IDSP portal function or Internet connectivity and preferred sending data by email over using the portal. However, the districts and number varied from one week to the other depending on availability of electricity, presence of data entry operators, and functional computers.

#### Data management

Few districts (44%) and facilities (15%) calculated the “reporting week” correctly (Monday to Sunday) despite the schedule been provided and made available on the IDSP portal. Majority (41%) of the facilities followed “Sunday to Saturday” schedule since they were expected to submit data latest by Monday morning to the DSU. About 85% districts accepted reports through the next week. The correct deadline (Tuesday) for district to state reporting was stated by 71% districts.

All subcenters photocopied S forms weekly. At the PHC/GMC/RH level forms were electronically filled and printouts taken. No additional costs for photocopies or printouts were available and therefore the staff hesitated to maintain office copies of the submitted forms. Office copies for 14% S forms, 32% L forms and 33% P forms were missing for the 13 weeks reviewed during data accuracy verification.

#### Data analysis

Although the facility registers recorded the date, age, location, and gender of the patient, IDSP reporting formats and portal entry system does not include these attributes in regular data. Line graphs were frequently available at the district levels for malaria (62%) than for acute diarrheal disease (29%) and syndromic fevers (23%). Thirty (88%) districts cited lack of time as the main reason for not performing disease trends for regular data.

#### Outbreak detection

Majority of the districts (76%) relied on syndromic data (S forms) as compared presumptive data (P forms) (59%) to detect unusual clustering or increase in the number of cases. At the facility level majority (30, 65%) used routine analysis of their own data to detect outbreaks. Few districts (23%) and facilities (22%) used lab confirmation (L forms) to detect outbreaks. In addition, 17 (50%) districts scanned media reports and 22 (64%) captured information from the community. Twenty- five (73%) districts maintained a rumour register and 22 (65%) updated it. The register was observed in four of the ten (40%) visited districts. Over 90% of the facilities visited did not maintain a rumour register. Majority of the districts (91%) and facilities (86%) prioritized proper case management over reporting to the next level indicating a preference for curative tendencies.

#### Outbreak/ epidemic preparedness and response

All districts had a rapid response team (RRT) and 24 (71%) had a clearly defined Epidemic Management Committee (EMC). Twenty seven (79%) districts had a written plan for response although few (23%) had evaluated it. Five of the 10 (50%) visited districts were able to provide a physical copy for verification. Fourteen (41%) districts had a method in place to forecast an outbreak of the 13 diseases based on institutional learning and analysis of previous data. Significant number of districts (88%) had access to emergency stocks of drugs and supplies at all times in past year which was confirmed in nine of the ten (90%) visited districts. Few districts (18%) experienced shortage of drugs, vaccines or supplies during the most recent outbreak. Twenty four (71%) districts had a clearly defined budget line or access to funds for outbreak response and half of them rated the amount as adequate. Twenty two (65%) districts stated that administrative delays made the funds less accessible despite availability. All districts requested clear guidelines for use of vehicles and fuel allowance during outbreaks. The Case Fatality Rates for the total outbreaks reported in the state during 2010 and 2011 for all diseases were within WHO acceptable limits.

**Figure 4:**
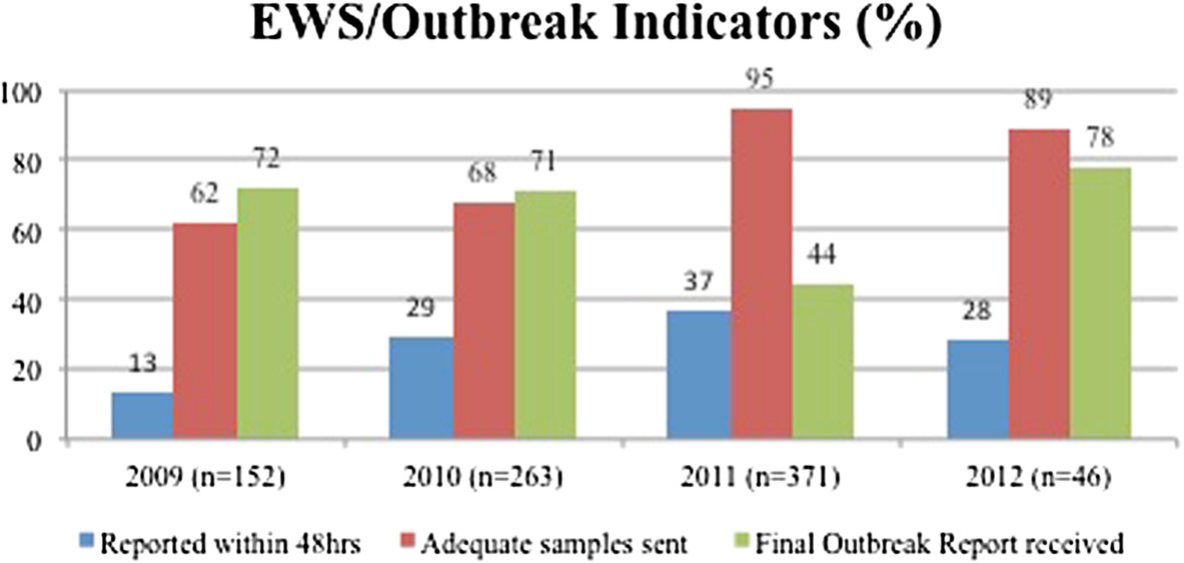
**Outbreak indicator performance of the system (2009–2012).**

Thirty-one (91%) districts reported a suspected outbreak in the last 6 months of which 24 responded within 48 hrs and all looked for risk factors. None of the three identified program outbreak indicators were met at 80% target but significant improvements have been recorded from 2009–2012 (Figure 4). Submission of the final outbreak report was the weakest. A majority of the districts (62%) and facilities (80%) had access to a written standard case management protocol. Four (40%) of ten visited districts and an equal number of facilities (41%) were able to provide a physical copy. All 34 districts reported use of outbreak data for action in the past year which included additional rounds of water purification, container surveys, health promotion and population awareness, stockpiling medications; preventive measures such as mass chemoprophylaxis for diphtheria and a doxycycline prophylaxis strategy in the district following increase in the incidence of leptospirosis in the last three years.

#### Feedback

No formal mechanism for feedback existed at any level. Periodic “Weekly Epidemiological Alerts” were disseminated by the SSU in 2010 but stopped once the epidemiologist proceeded on leave. Feedback from the state to the districts was significantly higher than the feedback from the districts to the facilities (*p* < .0001). Feedback was more frequently provided by email / official report (86%) followed by verbal communication (73%). Thirty facilities (83%) provided feedback to the communities and 18 (47%) provided evidence for it. Feedback at all levels was ad hoc; clustered around a suspected/ confirmed outbreak, not given to all RUs or districts and there was no specific schedule or budget identified.

### Support functions

#### Standards and guidelines

Surveillance manuals on the IDSP portal were not up-to date, difficult to understand and available only in English language. Medical officer’s manual developed under the IDSP was available in six (15%) of the 30 facilities and half of them were able to produce a physical copy for review. As per the manual a data tally sheet is to be maintained by the pharmacist within every facility- however this is not followed up at any level within the program. This sent mixed signals to the staff as to which part of the manuals was to be strictly followed and which not, which version is the latest and when these are updated or changed.

#### Laboratory capacity

There was a stark discrepancy in the tests performed and practices at the PHC, RH and RL levels (Table 3). Generally, at the PHC level only blood smear examination for malaria, Widal test for typhoid, and sputum smear examinations for tuberculosis were done. All other tests the patients were referred to private labs or to the District Public Health Laboratory (DPHL). Even the RLs did not conduct all tests as expected (Table 4) despite the provision of additional annual funds and equipment. Most peripheral lab staff were hired on a contractual basis and IDSP did not provide additional lab personnel or training at any level. Stringent procurement requirements have delayed equipment purchase at RL and non-provision of additional staff or incentives leads to overburdening of regular staff that refused to work for IDSP cases.

**Table 3:** Assessment of the laboratory structures [n (%)]

	**PHC (10)**	**RH (8)**	**Referral (7)**	**Total (25)**
Samples labelled adequately	10 (100)	7 (87)	6 (86)	23 (92)
Copies of reports available	10 (100)	5 (52)	2 (29)	17 (68)
Manual for test procedures	7 (70)	6 (75)	3 (43)	16 (64)
Temperature sheets (refrigerators, freezers and incubators)	0 (0)	1 (12)	4 (57)	5 (20)
Accurately labelled reagents	4 (40)	0 (0)	4 (57)	8 (32)
Accurate reagent expiry dates	6 (60)	0 (0)	4 (57)	10 (40)
Adequate gloves	8 (80)	7 (87)	6 (85)	21 (84)
Sharps container with disinfectant	4 (40)	5 (62)	2 (29)	11 (44)
Functional bio-waste disposal system	8 (80)	7 (87)	6 (86)	21 (84)

**Table 4:** Diagnostic tests performed at Referral Labs [n (%)]

**Disease**	**Test**	**Referral labs**
Cholera and other entero-pathogens	Culture	5 (71)
	Sensitivity	6 (86)
	Serotyping	4 (57)
Typhoid	Serological tests (TyphiDot/Widal) Typhoid	5 (71)
	Blood Culture	7 (100)
	Antimicrobial sensitivity (including isolate confirmation with specific antisera)	4 (57)
Bacterial meningitis:	Rapid latex agglutination test	1 (14)
	CSF examination-wet mount	7 (100)
	Gram stain and culture	7 (100)
	Antimicrobial sensitivity	7 (100)
Hepatitis A/E	IgM μ capture ELISA	1 (14)
Measles	IgM μ capture ELISA	1(14)
Dengue	IgM μ capture ELISA	4 (57)
Dengue	Antigen detection	1 (14)
Diphtheria	Smear examination	7 (100)
	Culture	5 (71)
Leptospirosis	ELISA	1(14)
	Rapid test	2 (29)
Chikungunya	National Institute of Virology Kits	1 (14)
Malaria	Rapid DT	1 (14)

#### Supervision

There were no guidelines on the required/expected number of supervisory visits at any level. Common reasons identified for inadequate and irregular supervisions were additional responsibilities at district (24, 70%); lack of funds (12, 35%); lack of staff at the DSU (11, 32%); not mandatory (9, 26%); and vehicle availability (30, 88%). A majority 16 (47%) districts and 24 (52%) facilities reported that a mandatory quarterly visit from the next level will improve reporting and provide much needed assurance that the work of peripheral staff was appreciated.

#### Training

Training was significantly higher at the district level compared to the facility levels (p < .0001). IDSP focal person at 17 (50%) districts had a degree in public health. In nearly half of the districts none of the subordinate staff was trained in integrated disease surveillance (Table 5). Often it was DSO and the epidemiologist who were trained in the two-week FETP course. Duration of training of the data entry operators was typically two days and the last training for health assistants and data managers at any level was conducted in 2006 despite 8% of the annual budget (approximately US$ 66,480 in 2011) being earmarked for it. The main reason identified by 23 (68%) districts for lack of trained staff was vacant state training consultant position and lack of an adequate training strategy at any level. Trained personnel were either transferred to another facility or have left jobs due to contractual positions (7, 21% districts). Other causes included lack of funds at the district level (19, 56%); and training not mandatory (13, 38%). No updated database of trained staff in position was available at the district or state headquarters, which made accurate estimations difficult. EDUSAT a satellite based audio-visual educational network established between the CSU and the 34 DSUs and selected tertiary medical teaching RUs aimed to cover training component. However, the equipment was installed in 40 of the 58 identified sites and was functional only in 31 sites.

**Table 5:** Human resources and training status

**Positions**	**Sanctioned**	**Filled**	**Trained**
**Epidemiologist**	36	22	20
**Microbiologist**	3	2	2
**Entomologist**	1	0	0
**Training Consultant**	1	0	0
**Finance Consultant**	1	1	1
**Data Manager**	36	22	21
**Data Entry Operator**	59	46	3
**Total**	137	93 (68)	47 (50)

#### Resources (financial, human, material)

Financial resources were adequately available in the state and an average of 46.14% under spending of sanctioned funds was observed over the last seven years. Despite which, at the state level forty four (32.2%) of the 137 sanctioned positions under the IDSP were vacant as of January 2011 (Table 5). Frequent transfer and turnover of staff at all levels are major hindrance for program progress. The State Surveillance Officer (SSO) position was an additional charge assigned to an Additional District Health Officer (ADHO) who also heads two other major state health programs.

Despite the availability of ear marked annual budgets no regular training or supervisory visits were conducted. Availability of logistic and communication resources were better at facilities than at district surveillance units (Table 6). In general most facilities were provided with express feeders under the NRHM. Only one of the subcenter’s with a new building did not have electricity. Availability of vehicles was a major issue in over 90% of the districts and unclear guidelines on spending available funds left it to the interpretation of the higher authorities for their use. Despite availability of vehicles, guidelines for their use and persons eligible for using them were unclear. Fuel charges had to be first borne by the DSO and epidemiologist and it took months for reimbursement. Secondly, unclear and non-standardized guidelines on the payment of Travel Allowance and Daily Allowance (TA/DA) proved detrimental for the motivation to conduct supervisory visits and outbreak investigations.

**Table 6:** Resources available at facilities and District Surveillance Units (DSU) respectively [n (%)]

	**Sub centre (16)**	**PHC (16)**	**RH (8)**	**GMC (6)**	**Facility total (46)**	**District total (34)**
**Logistics**						
24x7 Electricity	15 (94)	16 (100)	8 (100)	6 (100)	45 (98)	22 (65)
Inverter	5 (31)	14 (87)	8 (100)	2 (33)	29 (63)	16 (47)
Vehicles	-	16 (100)	6 (75)	2 (33)	24/30 (80)	6 (18)
**Data Management**						
Stationery	14 (87)	16 (100)	8(100)	3 (50)	41 (89)	27 (79)
Calculator	12 (75)	15 (94)	6 (75)	3 (50)	36 (78)	28 (82)
Printers	-	16 (100)	7 (87)	4 (67)	27/30 (90)	2 (76)
**Communication**						
Telephone service/	1 (6)	13 (81)	8 (100)	5 (83)	27/30 (90)	21 (62)
Fax	-	-	4 (50)	3 (50)	7/15 (50)	17 (50)
Computers with Internet	-	16 (100)	7 (87)	3 (50)	26/30 (87)	32 (94)
**IEC Materials**						
Posters	7 (44)	7 (44)	1 (12)	1 (17)	16 (35)	12 (35)
Megaphone	10 (62)	10 (62)	2 (25)	0 (0)	22 (48)	2 (6)
VCR and TV/Projector*	-	14 (87)	5 (62)	2 (33)	21/30 (70)	23 (68)
**Hygiene and sanitation materials**						
Spray Pump	0 (0)	0 (0)	6 (75)	2 (33)	8 (17)	7 (21)
Disinfectant	12 (75)	15 (94)	7 (87)	2 (33)	36 (78)	15 (44)
Protection Material	13 (81)	15 (94)	7 (87)	2 (33)	37 (80)	10 (29)

*VCR and TV set at facility/ Projector at district.

#### Coordination

Minutes/reports of the co-ordination committee were available at four (40%) of ten visited districts and eight (17%) facilities.

## Discussion

The implementation of IDSP in all 34 districts of the state although encouraging, is only partially satisfactory. Significant progress has been made but gaps remain and the current transition of the system offers a unique opportunity to implement the necessary structural changes and should be exploited to its potential. The use of the Standard Case Definitions (SCDs) was poor at the periphery hinting that the syndromic SCDs need clarifications. All SCDs should be made available in Marathi language instead of the current “only English” versions in order to improve their use [27,35]. Further, the reporting deadlines and definition of a reporting week were poorly understood affecting reporting quality. Annual circulation of job aids with SCDs and reporting deadlines should be implemented to improve data collection and reporting as demonstrated from studies in Tanzania, Mozambique, Uganda and Ghana [36-40].

Case registers were more complete for IPD patients and at the peripheral level, a finding similar to other studies from Mozambique and Uganda [39,41]. Lack of reporting formats and office copies of reports sent thereof compromised data verification and institutional learning. Staff at periphery printed or used photocopies at their own costs. At state level, storage and distribution of large quantities of printed formats was a logistic problem and therefore provisions in local contingency funds should be made at district/facility levels for printing them. Alternately, printed IDSP registers should be considered at all levels to ensure adequate standardization and documentation at source [27,42-44]. Second, the system does not currently collect data on disease mortality which should be addressed in the next restructuring [45].

Weak lab infrastructures at periphery compromise regular and outbreak surveillance functions and have been reported earlier from Tanzania, Ethiopia and South Sudan [2,6,46]. The PHCs are the first contact for diagnostics in rural areas, and 37% of them did not have a lab facility in our study. Further, only half of the districts could confirm all the priority diseases. Sample collection and transport was the weakest for stool samples, even when the maximum outbreaks and the burden from diarroheal diseases remained high in every district. Staff in referral labs was hesitant in processing samples from periphery due to inadequate, inappropriate or contaminated samples. Availability of transport media and cold chain boxes for sample transport needs immediate attention even in the light of vaccination programs such as polio. Annual cold chain audits should be considered for regular monitoring [47]. Scaling up the use of rapid diagnostic tests to manage laboratory shortcomings at periphery should considered [48].

The referral network of ten labs established in the state through IDSP funding although a step, is sub-optimally efficient, given that four to five districts are covered under each RL. Second, the network is envisioned primarily for outbreak functions leaving regular laboratory functions still weak. A parallel strategy should be to strengthen the District Public Health Laboratories (DPHL) available at each district which currently covers regular surveillance function for IDSP. Capacity building in terms of personnel and equipment at each level should be planned consequently through core state budgets to strengthen the states laboratory infrastructure for *both* regular and outbreak functions [49,50].

Electronic data processing is a major advantage in surveillance [51]. However, the state fails to reap optimum benefits due to interrupted Internet services, unstable IDSP portal, poor staff training and lack of data entry operators. Currently all RUs send paper forms to the DSU and the data volume significantly overburdens the staff. Through NRHM, two computers, broadband Internet services, and a data entry operator have been provided to a majority of the PHCs [52]. Secondly, under the Indian Public Health Standards increasingly PHCs that operate 24X7 are being provided with express electricity feeders [53]. Shifting IDSP data entry progressively from DSUs to facility level is therefore pragmatic for several reasons. One it is equipment and personnel wise more feasible now than before, it eliminates the logistics, costs and burden of paper based reporting to district level, improves data quality by sheer reduction in data volume, speeds up surveillance which can support the installation of automated outbreak and aberration detection mechanisms within the system, and allows the DSU more time to analyze the data as the burden of data entry is taken away.

Weak data analysis at every level was observed on our study as from those in Ghana, Lesotho, Tanzania, Uganda and in other states of India [7,35,42,50,54-57]. The staff at the DSU is better trained and receives frequent feedback from the SSU as compared to facilities giving them a position of advantage for data analysis. However, a lack of logistic resources and the burden of data entry prevent them from applying their skills optimally. Developing clear guidelines for data entry, management and analysis at each level should be considered [2]. Additionally, regular in-service training supported by adequate supervision of the surveillance staff at all levels should be incorporated [39].

Irregular and ad hoc feedback was observed at all levels in our study. Feedback at facility level was better than that at the DSU level and reason identified included frequent change in the SSO. At the facility level, routine monthly review meetings conducted by the block health officers, medical officers meetings and quarterly subcenter staff meetings served as a platform for IDSP review but differed from one district to the other. Feedback is an essential component for maintaining involvement and motivation of surveillance staff [38,56,57]. Formal mechanisms should be developed with accurate guidelines for frequency and components of feedback at all levels (essentially on a quarterly basis for DSUs) including budgeting for supervisory visits [4].

Training is significantly associated with data analysis, feedback and supervision [58,59]. Training of DSO and epidemiologists was better than that of the peripheral staff in our study like in several others [37,60]. Lack of adequate training schedule, funds, discrepancy with regards to state or district responsibility and vacant state training consultant position affected the availability of trained personnel in our study. Institutionalizing training for integrated disease surveillance in regular medical and paramedical curricula is considered the most sustainable strategy and should be incorporated [4]. Practical on-site in-service trainings for surveillance and lab staff should be mandatorily planned as an annual activity [59,61]. High attrition of trained staff was another reason for lack of trained personnel in our study including others in Ethiopia and Lesotho [46,50]. A detailed updated database of trained personnel should be maintained at district level and trainings should be coordinated by districts rather than the state to allow efficient overview [27,49].

Outbreak detection and response capacity, and rightly so, was better at the periphery compared to the DSU level. The peripheral staff had better understanding of the outbreak thresholds, was well supervised by the THOs, and had improved access to vehicles and logistic support as compared to the DSUs. Adequate supplies were observed at both the district and facility levels in our study indicating sufficient resources. Heavy focus on curative approaches and access to treatment manuals ensured proper case management and reductions in case fatality rates. As first responders are always the peripheral staff, all factors, to the advantage of the system, worked towards significantly improving outbreak response [8].

Some of the limitations included the fact that weak data management and analysis did not allow automated outbreak detection despite the availability of electronic data. This should be instated as the next step in improving the system structure [62]. Outbreak indicators were well understood and implemented by peripheral staff, however poor filing of final outbreak reports jeopardized documentation and hence the assessment of response adequacy and institutional learning. A general tendency to under-report the total number of outbreaks was observed due to fear of action from the higher authorities. Supportive supervision with compulsory monitoring of identified IDSP indicators should be developed at each level to promote collective responsibility and avoid finger pointing [27,36,63,64].

Availability of sustainable resources (human, logistic and equipment) are at the root of surveillance performance [65]. Thirty per cent IDSP positions were vacant in the state despite availability of financial resources leading to multiple responsibilities on existing staff including the SSO and the DSOs. A finding commonly reported from several other low and middle income countries [66]. Further the SSO and DSOs were selected ad-hoc, frequently transferred and saw surveillance as an additional burden. Contractual temporary positions are the main reason for high turnover of lab and IDSP staff in our study. Lack of job security, uneven and unrevised salary structure, with administrative delays in processing contracts and monthly pays demotivated district and facility staff to take on or continue IDSP positions (Personal communication with SSO). Retaining trained staff although a challenge is of utmost importance [36,43]. Developing permanent cadre of skilled surveillance personnel holds the key to program viability and should be actively pursued [67].

Competent staff should be backed up with appropriate and adequate logistic and equipment support for effective communication, laboratory function and data management [6,41,48]. Currently, the IDSP provides annual procurement costs only for referral laboratories and none for rapid diagnostic testing at peripheral levels which should be re-considered. Although adequate communication equipment was in principle available, the availability at district level was weaker as compared to the facility levels. One time procurement of hardware/software for data management was made in the initial phases of the program. However, most equipment is now old, poorly maintained and requires repair or replacement. Effective communication systems for data transmission determine speed and completeness of reporting and provision of annual maintenance contracts for purchased equipment should be envisaged in annual budgetary planning [50,51].

Availability of transport vehicles was weakest at sub-centre level where staff is expected to perform active syndromic surveillance on a bi-weekly basis. Chronic shortage of staff in the backdrop of rapid population growth has resulted in dis-proportionate personnel and facility distributions making active surveillance a horrendous task [68]. Geographical areas are impossible to cover on foot and providing interest-free two-wheeler loans to sub-centre staff is suggested. ASHA (Accredited Social Health Activists) identified at the village level have been included in the IDSP to overcome some of these challenges and a minimal monetary compensation is offered for every outbreak reported. However, this arrangement is not fully exploited to its potential in all districts and delays in payments have affected the inclination of ASHA workers to participate. Additionally, active involvement and community interest should be sustained by organizing regular feedback meetings which less than half of the facilities were implementing in our study [57].

Vehicle availability was problematic in majority of the DSUs in our study hindering supervision and outbreak investigations. Similar findings have been reported from IDS assessments in Iraq and Nigeria [37,69]. IDSP designated vehicles should be made available on priority basis and clear guidelines on the use of vehicle contingency funds within the program should be developed.

Weak inter-sectorial and inter- programmatic coordination was observed in our study despite availability of a designated focal person at all levels in majority of the districts in our study like in others from Uganda, Ghana, Ethiopia, other states in India and Mali [35,46,57,58,70]. Additionally, poor documentation of review meetings made its functionality rather doubtful. In order to avoid that the IDSP becomes another vertical disease surveillance program and to eliminate existing lacunae as identified in our study we suggest that the DSUs should be established as permanent structures within the district health care system in Maharashtra. Structural integration of the IDSP will allow gradual progressive channelling of surveillance activities of all major vertical disease control through these units resulting in effective coordination [36,63,70]. Further, instead of providing IDSP as an additional charge to existing ADHOs who overlook multiple programs, separate *full time positions* for SSO and 34 DSOs should be created. An ideal strategy would be to recruit existing contractual epidemiologists on long term or permanent basis. Given that the salaries of these officers were borne by the state since 2005, resources are not an issue, but the policy is!

## Conclusion

In conclusion, the findings of the study confirm that the IDSP in Maharashtra has made satisfactory improvements on a majority of core and support surveillance functions. Chief amongst them are the development of standard case definitions, laboratory manuals and adequate outbreak response. However, data management and analysis were weak at all levels. We note that all support functions were key to the performance of core surveillance functions. Specifically:

•Case and outbreak detection depended heavily on definitions and job aids developed and on the knowledge and skills of surveillance personnel

•Case confirmation was dependent on laboratory infrastructure and the knowledge and skills of laboratory personnel

•Case notification was dependent on communication and logistic equipment available at every level

•Data management and analysis was dependent on communication equipment and the knowledge and skills of surveillance personnel

•All of which were primarily affected by three things

•Availability of adequate human resources

•Training of surveillance and laboratory staff

•Supervision

•Feedback

It is essential to note here, that although laboratory functions; feedback and training were weak at the peripheral levels, a majority of the logistic and equipment issues; and more importantly coordination was weak at the district levels. Strengthening each aspect is unrealistic. As a first step, the support functions (except financial resources) need to be targeted for improvements.

The average performance of the IDSP, despite a well-designed structure and the availability of adequate financial resources indicate that certain other barriers hinder its optimal functioning. Incomplete adoption of the original project implementation plan, which theoretically addresses a majority of the shortcomings identified in our assessment, is probably the main amongst them and incomplete structural integration of the IDSP within the state health service system the other. Given that the IDSP is now in transition from an externally funded project to an independent core central program in the state, structural alignments at both ends- IDSP itself and the state health service system which receives it are necessary. Chief amongst them is establishing the state and district surveillance units as permanent structures within the state health infrastructure in order to avoid the integrated disease surveillance system from ending up isolated! Improving surveillance quality should be the next on the agenda for the state [71].

## Competing interests

The authors declare that they have no competing interests.

## Authors’ contributions

RP designed the study, conducted the fieldwork, analyzed data and drafted the manuscript. PA and SShardul supervised the field study, assisted with data access, and provided inputs to the manuscript. NA and SV were responsible for the double data entry, randomized data quality checks and assisted with descriptive analysis. SShukla assisted with the statistical analyses and review of the manuscript. MM supervised the study, assisted with analysis and contributed to the manuscript. All authors read and approved the final manuscript.

## Authors’ information

RP has completed her Masters in International health from Humboldt University in Berlin and is currently a doctoral fellow at the Institute of Public Health, University of Heidelberg, Germany. PA is a MD and works as the State Surveillance Officer within the IDSP in Maharashtra, India. NA has completed her Post Graduate Diploma and works as the Data Entry Officer within the State Surveillance Unit of the IDSP. SV is a trained statistician working at the Chest Research Foundation in Pune, India. SShukla is a statistician by training and works as faculty at the Centre for Modelling and Simulation, University of Pune, Pune, Maharashtra, India. SShardul is MD in Preventive and Social Medicine and works as the state epidemiologist at the IDSP State Surveillance Unit, Maharashtra, India. MM is a MD with Diploma in Tropical Medicine and Public Health and works as an Assistant Professor at the Institute of Public Health, University of Heidelberg, Germany and supervised RP for her doctoral research.

## Pre-publication history

The pre-publication history for this paper can be accessed here:

http://www.biomedcentral.com/1471-2458/13/575/prepub

## Supplementary Material

Additional file 1 **List of diseases and syndromes covered under each type of surveillance.** Describes the 21 syndromes and diseases covered under syndromic, presumptive and lab confirmed routine surveillance within the IDSP. Click here for file
