# Supplementary material for: Assessment of the core and support functions of the Integrated Disease Surveillance system in Maharashtra, India
Source: BMC Public Health. 2013 Jun 13;13:575. doi: 10.1186/1471-2458-13-575 (PMC3693947; doi:10.1186/1471-2458-13-575)
Supplement: Additional file 1 — List of diseases and syndromes covered under each type of surveillance. Describes the 21 syndromes and diseases covered under syndromic, presumptive and lab confirmed routine surveillance within the IDSP. [file 1471-2458-13-575-S1.docx]

| **Syndromic (S form)** | **Presumptive (P Form)** | **Lab Confirmed (L form)** |
| --- | --- | --- |
| 1. FEVERS  - only fever - fever with rash, - fever with bleeding - fever with daze, semi-consciousness or unconsciousness  1. COUGH   Cough or without fever   - less than 3 weeks - more than 3 weeks  1. DIARROHEA   Loose watery stools of less than 2 weeks duration   - with some or much dehydration - with no dehydration - with blood in stools  1. JAUNDICE   Acute jaundice cases < 4wks   1. AFP   Acute Flaccid Paralysis (less than15yrs)   1. OTHERS* ( Neonatal Tetanus) | 1. Acute Diarrhoeal Disease (including acute gastroenteritis) 2. Bacillary dysentery 3. Viral hepatitis 4. Enteric fever 5. Malaria 6. Dengue / DHF /DSS 7. Chikungunya 8. Acute encephalitis syndrome 9. Meningitis 10. Measles 11. Diphtheria 12. Pertusis 13. Chickenpox 14. Pyrexia of unknown origin (PUO) 15. Acute respiratory tract infection(ARI) or influenza like illness 16. Pneumonia 17. AFP <15 years of age 18. Dogbite 19. Snakebite 20. Leptospirosis 21. Unusual syndromes not captured above ( Neonatal Tetanus) | 1. Dengue/DHF/DSS 2. Chikungunya 3. Japanese encephalitis 4. Meningococcal meningitis 5. Typhoid fever 6. Diphtheria 7. Cholera 8. Shigella dysentery 9. Viral hepatitis A 10. Viral hepatitis E 11. Leptospirosis 12. Malaria 13. Other (Neonatal Tetanus) |

Annex: List of Diseases and Syndromes covered under each type of surveillance
